# Unexpected retraction of distal cut end of flexor pollicis longus tendon

**DOI:** 10.4103/0974-2700.50755

**Published:** 2009

**Authors:** Rajinder Kumar Mittal, Ramneesh Garg, Ashish Gupta

**Affiliations:** Department of Plastic Surgery, Dayanand Medical College and Hospital, Ludhiana-141 001, Punjab, India

Sir,

We are presenting a case with unusual presentation of the distal cut end of flexor tendon (flexor pollicis longus, FPL). Any patient presenting with laceration anywhere over the hand has to be assessed preoperatively for possibility of injury to either bone or soft tissue underneath. Among soft tissues, tendon, vessels and nerves have to be looked for. For vessel injury, distal vascularity is checked and for nerve injury; sensations distal to the laceration are assessed. Injury to the tendons results in loss of joint movements of the respective digits. A careful preoperative examination helps the surgeon in identifying various structures.

**Figure 1:**
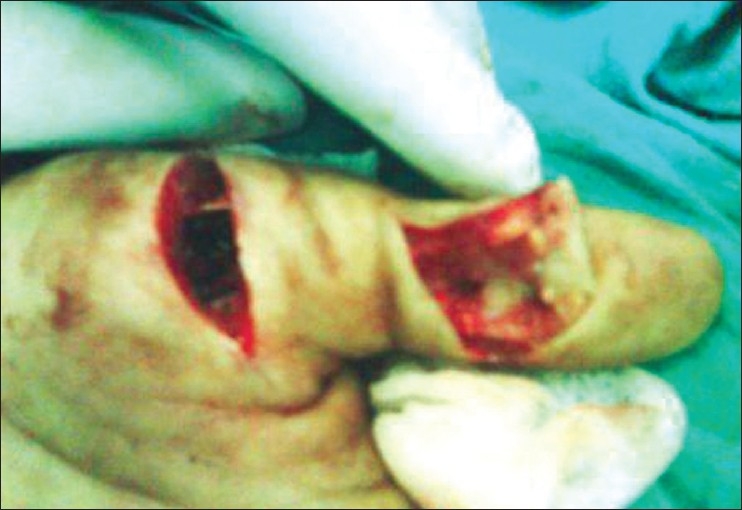
Laceration at the base of thumb and a distal surgical exposure showing base of distal phalanx and no visibility of FPL

**Figure 2:**
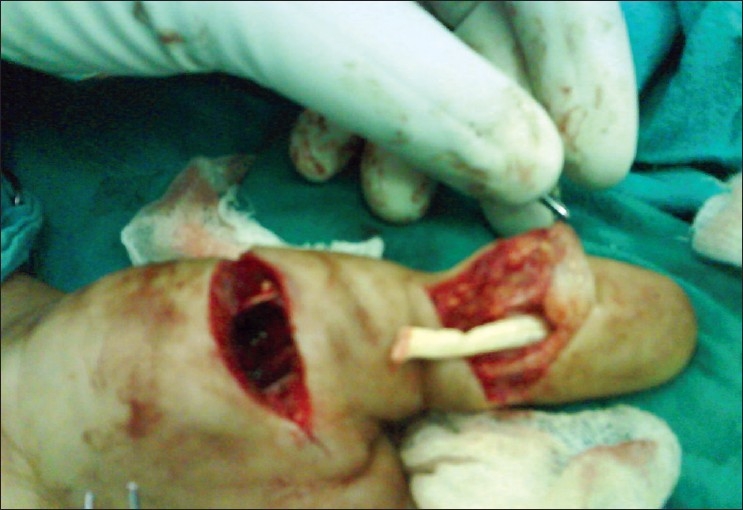
Distal cut end of FPL retrieved from pulp of the thumb

In any flexor tendon cut injury, the proximal cut end is known to retract and may need proximal incision for the retrieval of tendon.[1] Literature is full of various methods to retrieve the proximal cut end of flexor tendons. Distal cut ends of flexor tendons shift distally if injury happens to be while the fingers or thumb are in flexion. These distal cut ends usually lie in the tendon tunnel only and are retrieved through the laceration itself by flexing the fingers. There is no need for a separate distal incision. Contrary to this routine, we had a case with a 3-hour-old history of laceration at the base of thumb, i.e., just proximal to the metacarpophalangeal joint of thumb. Thenar muscles were cut and flexion at interphalangeal joint of thumb was not there. So clinical diagnosis of the laceration of FPL tendon was made. On exploration of the laceration, we could not retrieve the distal cut end of FPL even after flexion of interphalangeal joint repeatedly. Then a distant distal horizontal incision was made over the IP joint, but the distal cut end was still not visible [Figure 1]. For once thought came about possibility of congenital absence of FPL, which is a rarity.[2] Incision was further extended over the pulp obliquely. Approximately 3-cm-long cut end was lying curled up in the pulp tissue [Figure 2] with the cut end pointing towards the tip. Even the insertion of FPL was distal to the expected site, which is normally at the base of distal phalanx.[3] According to us, sudden extension of the interphalangeal joint of the thumb, as withdrawal movement after injury, led to the severe retraction of distal cut end of FPL into the pulp of the thumb. The retrieval of the tendon was not possible because the direction of the free end of the distal cut end of tendon was pointing towards tip of the thumb instead of towards the laceration itself. This case was a learning experience for us that even the distal cut end of flexor tendon can retract so much that its retrieval becomes difficult. Change in the direction of free end of the distal cut end of tendon should also be kept in mind.
